# Sphingosine 1-Phosphate (S1P) Induced Interleukin-8 (IL-8) Release Is Mediated by S1P Receptor 2 and Nuclear Factor κB in BEAS-2B Cells

**DOI:** 10.1371/journal.pone.0095566

**Published:** 2014-04-17

**Authors:** Michael J. O’Sullivan, Nobuaki Hirota, James G. Martin

**Affiliations:** Meakins-Christie Laboratories, Department of Medicine, McGill University and McGill University Health Centre Research Institute, Montréal, Canada; National Jewish Health, United States of America

## Abstract

The airway epithelium may release pro-inflammatory cytokines and chemokines in the asthmatic airway. Sphingosine 1-phosphate (S1P) is a bioactive lipid, increased in the airways of asthmatics, that may trigger the release of the potent neutrophil chemoattractant Interleukin-8 (IL-8) by epithelial cells. S1P is a ligand for 5 G protein-coupled receptors, S1PR1-5. We wished to explore the mechanisms of S1P induced IL-8 secretion with regard to the receptor(s) and downstream signaling events involved. Our results indicate that S1P induced IL-8 release is mediated by S1PR2 and the transcription factor NF-κB. Since the Epidermal Growth Factor Receptor (EGFR) and reactive oxygen species (ROS) have been implicated in IL-8 release in response to activation of other G protein-coupled receptors, we examined their importance in S1P induced IL-8 release and established that they are not involved. This study reveals S1PR2 and NF-κB as potential therapeutic targets in neutrophilic airway diseases such as severe asthma.

## Introduction

S1P is a bioactive lipid important in immune system regulation, angiogenesis, migration and proliferation [1]–[4]. S1P is produced when sphingosine, derived from ceramide, is phosphorylated by sphingosine kinase I or II. There are currently five known S1P receptors (S1PR1-5) and these are G protein-coupled. S1P binding to these receptors can elicit diverse signaling mechanisms owing to the heterogeneity of these receptors and their coupling to different G proteins. Synthesis of S1P occurs in many cell types including platelets and mast cells [5], [6]. S1P has been shown to be increased in the bronchoalveolar lavage of asthmatics upon segmental allergen challenge when compared to healthy control subjects [7]. S1P has also been shown to induce contraction of airway smooth muscle cells, strengthening its potential role as an important lipid mediator in the asthmatic airway [8].

Neutrophils, among other leukocytes, play an important role in asthma pathogenesis. When compared to healthy control subjects, severe asthmatics possess more neutrophils in the induced sputum [9]. It is well established that neutrophils undergo chemotaxis towards an increasing gradient of the chemokine interleukin 8 (IL-8), as reviewed by Baggiolini et al. [10]. IL-8 release from structural cells in the lung is therefore a possible avenue by which neutrophil recruitment occurs in the asthmatic airway.

S1P has previously been shown to induce IL-8 release from airway epithelial cells in a phospholipase D dependent manner [11], [12]. We wished to explore which S1P receptor(s) are involved in S1P induced IL-8 release from airway epithelial cells, as receptor inhibition could reveal novel therapeutic targets for the treatment of severe asthma. Transactivation of the EGFR is a requirement for leukotriene D4 (LTD_4_)-induced release of IL-8 from airway epithelial cells [13]. LTD_4_ also induces transactivation of the EGFR in airway smooth muscle cells and this phenomenon is dependent on the generation of ROS [14]. Because LTD_4_ is an agonist of the cysteinyl leukotriene Receptors 1 and 2 (CysLTR1/2) and these receptors are G protein-coupled, we hypothesized that S1P may mediate IL-8 release via ROS dependent transactivation of the EGFR and explored this hypothesis in the context of the airway epithelium. Finally we examined the role of IL-8 transcription factors in the process of S1P induced IL-8 release.

## Materials and Methods

### Reagents

W 123 (10 µM), JTE 013 (1–10 µM), CAY 10444 (10 µM), specific inhibitors of S1PR1, S1PR2, S1PR3 respectively, S1P (0.1–10 µM), and the EGFR inhibitor tryphostin AG-1478 (0.3–3 µM) were all obtained from Cayman Chemical (Ann Arbor, MI, USA). Helenalin (1 µM), inhibitor of NF-κB, pEGFR antibody (p-Tyr-845 SC-23420-R) and total EGFR antibody (SC-03) were obtained from Santa Cruz Biotechnology (Santa Cruz, CA, USA). SR 11302 (1 µM), inhibitor of activator protein-1 (AP-1) and SEW 2871 (10 µm), agonist of S1PR1 were obtained from Tocris Bioscience (Bristol, UK). Dichlorodihydrofluorescein diacetate (DCFH-DA) (10 µM) and N-acetyl cysteine (NAC) (1 mM) were obtained from Sigma-Aldrich (St. Louis, MO, USA). Luciferase reporter lysis buffer was obtained from Promega (Madison, WI, USA). GM6001 (25 µM), the broad-spectrum hydroxamic acid inhibitor of matrix metalloproteinases (MMPs) and TAPI-1 (10 µM), inhibitor or MMPs and tumor necrosis factor-α converting enzyme (TACE) were obtained from Calbiochem (La Jolla, CA). Fura-2 AM (10 µM), and pluronic F127 (0.02%) were obtained from Life Technologies (Carlsbad, Ca).

### Cell Culture

Human BEAS-2B cells (ATCC, Manassas, VA) were grown in DMEM:F12 10%FBS 100 U/ml penicillin, 100 µg/ml streptomycin and 2500 ng/ml amphotericin B (PSA) (Invitrogen, Carlsbad, CA, USA) in 75 cm^2^ tissue culture flasks at 37°C and 5% CO_2_. Culture medium was changed every 2 days and cells were seeded into new flasks when approximately 80% confluent. Cells were detached by incubation with 0.25% trypsin (Sigma-Aldrich). For experimentation, cells were seeded in 6 well plates at a density of 50 000 cells per well and grown for 3 days in culture medium. Cells were serum-starved for 24 hours in DMEM:F12 0.1%BSA (Sigma-Aldrich) with PSA. Starvation medium was changed prior to all experiments and culture supernatant was collected at the end of the incubation period.

For NF-κB luciferase reporter assays, cells were grown in DMEM 10%FBS 100 U/ml penicillin, 100 µg/ml streptomycin and hygromycin B. Starvation medium (CnT-17 basal medium) (CellNTec, Bern, Switzerland) was not changed prior to S1P stimulation. For intracellular calcium measurements, primary human airway smooth muscle cells obtained from lung transplant donors were cultured in DMEM 10%FBS with PSA and used between passages 3 and 5.

### Measurement of IL-8

IL-8 concentration in the culture supernatant was measured after a 4 hour incubation, with or without S1P, and respective inhibitors by ELISA using the CXCL8 Duoset (R&D Systems, Minneapolis, MN, USA).

### Measurement of ROS

Cells were seeded in dark-walled 96 well plates at a density of 10 000 cells per well in starvation medium for 24 hours. The cells were washed with Hanks balanced salt solution (HBSS) (137 mM NaCl, 4.2 mM NaHCO_3_, 10 mM glucose, 3 mM Na_2_HPO_4_, 5.4 mM KCl, 0.4 mM KH_2_PO_4_, 1.3 mM CaCl_2_, 0.5 mM MgCl_2_, 0.8 mM MgSO_4_, 5 mM HEPES) and incubated with fresh HBSS containing 10 µM 2′,7′-Dichlorofluorescein diacetate (DCFH-DA) (Sigma-Aldrich) for 30 minutes. Cells were washed with fresh HBSS and the baseline fluorescence intensity was read using a fluorescent plate reader (Tecan iControl, Männedorf, Schweiz, Switzerland) (Excitation = 485 nm, Emission = 530 nm). The cells were stimulated with S1P 1 µM or vehicle and fluorescence intensity was read every 5 minutes for 1 hour.

### EGFR Knockdown by siRNA Transfection

BEAS-2B cells were seeded in 6 well plates at a density of 25 000 cells per well in DMEM:F12 10%FBS (Invitrogen) without antibiotics. 12 hours later the medium was aspirated and 9 pmols of scrambled (SC-37007) or EGFR specific (SC-29301) siRNA with 2 µl of siRNA transfection reagent (SC-29528) in 1 ml transfection medium (SC-36868) was added to the cells for 5 hours. (Santa Cruz Biotechnology, Santa Cruz, CA, USA) 1 ml of DMEM:F12 20%FBS with PSA (Invitrogen) was added and the cells were incubated for another 18 hours. Medium was changed into DMEM:F12 10%FBS with PSA (Invitrogen) for 24 hours and cells were serum starved in DMEM:F12 0.1%BSA with PSA (Invitrogen) for 24 hours. Starvation medium was replaced and cells were stimulated with 1 µM S1P or vehicle for 4 hours.

### Western Blot

Cells were washed with ice-cold PBS (Invitrogen) following experimentation and lysed with ice-cold protein extraction buffer containing 50 mM TrisHCl (pH 8), 150 mM NaCl, 1% NP-40, 0.5% sodium deoxycholate, 0.1% SDS. Cell lysates were centrifuged at 13 000 RPM for 3 minutes and total protein supernatant was collected for SDS-PAGE (8%). 20 to 30 µg of protein diluted in distilled water and loading dye were boiled together for 6 minutes and equal volumes of sample were loaded onto the separating gel. After separation, proteins were transferred to a PVDF membrane (BioRad, Hercules, CA, USA) for immunoblotting. The membrane was blocked for 1 hour at room temperature in a 2% BSA in Tris-buffered saline (TBS). Membranes were incubated with primary antibody (total-EGFR) diluted 1∶2000 in TBS containing Tween-20 (TBST) overnight at 4°C. Membranes were incubated with secondary antibody (goat, anti-rabbit-HRP) diluted 1∶5000 in TBST for 1 hour at room temperature. Western blots were quantified using the ECL Plus Western blotting detection system (GE Healthcare, Little Chalfont, UK).

### NFkB Luciferase Reporter Assay

BEAS-2B cells were stably transfected with a plasmid containing a κB (GGGGACTTTCC) response element upstream of a hygromycin B resistance gene and a firefly luciferase construct. These cells were seeded into 24 well plates at a density of 50 000 cells per well in DMEM 10% FBS hygromycin B for 24 hours. Cells were serum starved overnight in CNT-17 basal medium (CellNTec) and stimulated with 1 µM S1P for 4 hours. Other cells were pretreated for 30 minutes with 1 µM JTE-013 and then stimulated with 1 µM S1P for 4 hours. Reporter lysis buffer (Promega, Madison, WI, USA) was used to lyse the cells. Whole cell lysates were collected and spun at 13 000 RPM for 3 minutes. 10 µl of supernatant was transferred to a 96 well plate for reading in the Tecan iControl luciferase system.

### Measurement of Intracellular Calcium

Human airway smooth muscle cells were seeded onto sterilized glass cover slips at a density of 25 000 cells per well in 6 well tissue culture plates in DMEM 10% FBS with PSA (Invitrogen). After one day, cells were serum deprived in DMEM 0.5% FBS 1%PSA medium for 3 days before analysis of intracellular calcium responses to 1 µM S1P using 10 µM Fura2-AM. To mediate loading of the Fura-2 AM, pluronic F127 (0.02%), along with the 10 µM Fura-2 AM was dissolved in HBSS for 30 minutes at 37°C. Any unloaded Fura-2 AM was washed out with HBSS. Cover slips were loaded into a Leiden chamber (Medical Systems, Greenville, NY) and an inverted fluorescent microscope with a 40X oil-immersion objective (Olympus, Tokyo, Japan) was used to measure signals emitted at 510 nm using a CCD camera (CoolSnapPro; Media Cybernetics, Bethesda, MD) controlled by Image Master software (Photon Technology International, Birmingham, NJ). Data was acquired as previously described [15].

### Statistical Analysis

Statistical analysis was carried out in the GraphPad Prism 5 software (GraphPad Software, Inc., San Diego, CA, USA). All data are expressed as means +1SE, with ≥3 independent observations per experiment. To test for statistical differences, one-way ANOVA with Tukey’s post hoc test was applied to experiments with ≥2 groups. For experiments with only 2 groups, we applied Student’s unpaired T-test. For comparison of fluorescence intensity curves, baseline values were normalized prior to S1P stimulation and a repeated measures ANOVA was applied. P values <0.05 were considered to be significant.

## Results

### S1P INDUCES IL-8 Release

**Figure 1:**
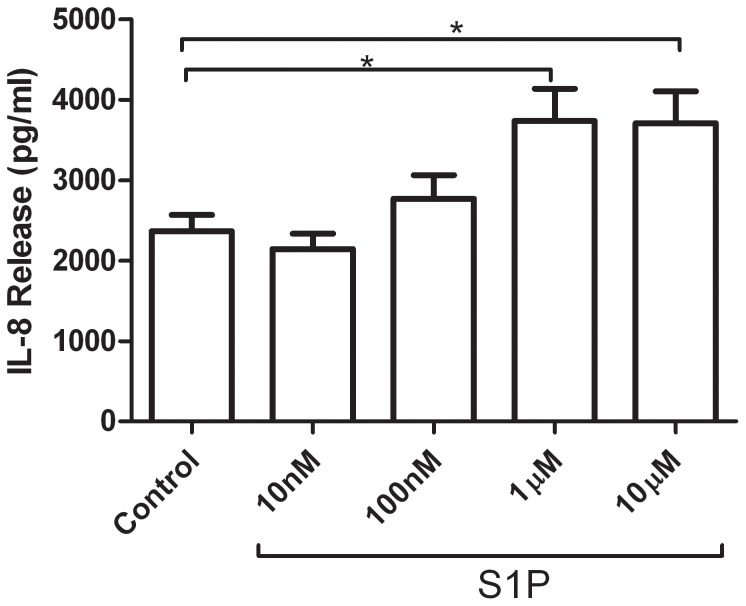
S1P induces IL-8 release in BEAS-2B cells. BEAS-2B cells were stimulated with various concentrations of S1P for 4 hours. Culture supernatant was analyzed for concentration of IL-8 by ELISA (n = 5). Data are representative of means+SE. ANOVA with Tukey post hoc pairwise comparisons. *P<0.05.

To confirm that S1P induces IL-8 release from BEAS-2B cells, the cells were incubated with various concentrations of S1P for 4 hours. ELISA analysis of the culture supernatant showed a dose-dependant increase in IL-8 release with S1P stimulation that was significant at a concentration of 1 µM (Fig. 1). For subsequent experiments, 1 µM S1P was used to stimulate IL-8 release.

### S1P Induced IL-8 Release is Mediated by S1PR2

**Figure 2:**
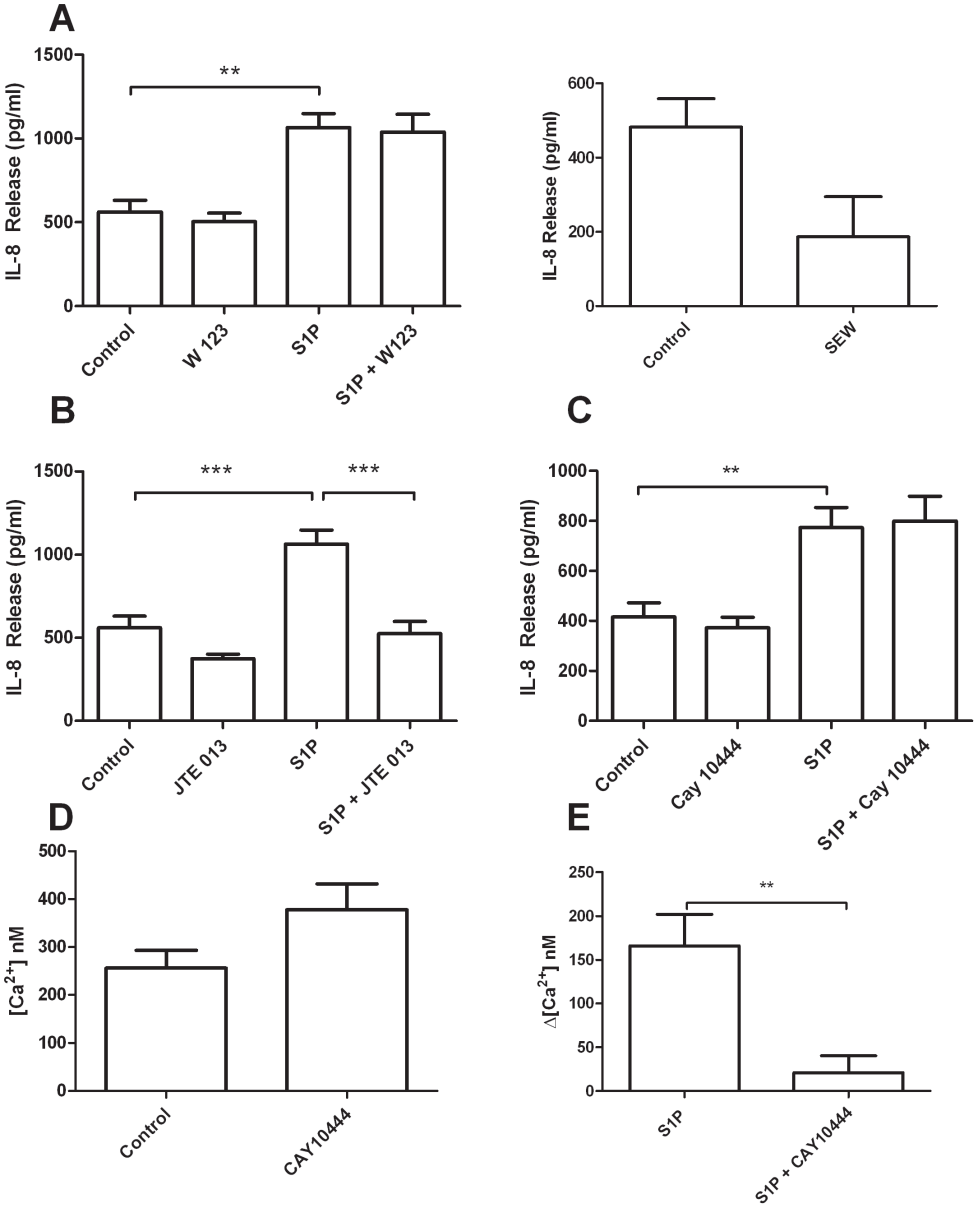
S1P induced IL-8 release is mediated by S1PR2 in BEAS-2B cells. BEAS-2B cells were pretreated for 30 minutes with (A) W 123 (n = 5), (B) JTE 013 (n = 5) or (C) CAY 10444 (n = 8), specific inhibitors of S1PR1, S1PR2 and S1PR3 respectively and then stimulated with S1P or vehicle for 4 hours. Culture supernatant was analyzed for IL-8 concentration by ELISA. Human airway smooth muscle cells were loaded with 10 µM Fura 2-AM, then treated with CAY10444 or vehicle (n = 5) for 30 minutes and intracellular calcium was measured by ratiometric fluorescence microscopy (E). Cells were stimulated with S1P (n = 5) and increases in resting intracellular calcium were recorded (F). Data are representative of means+SE. ANOVA with Tukey post hoc pairwise comparisons. **P<0.01, ***P<0.001.

To determine if S1P induced IL-8 release from airway epithelial cells is mediated by a specific S1P receptor, or group of receptors, BEAS-2B cells were pre-treated with specific S1P receptor inhibitors for 30 minutes prior to stimulation with S1P. W123, a competitive antagonist of S1PR1 (K_i_ = 0.69 µM), JTE-013, a selective antagonist of S1PR2 (IC_50_ = 17 nM), and CAY10444, a selective antagonist of S1PR3 (IC_50_ = 4.6 µM) were used as inhibitors of their respective receptors. Pre-treatment with JTE-013, but not with W123 or CAY10444, significantly inhibited S1P induced IL-8 release (Fig. 2 A). To ensure that S1PR1 and S1PR3 were not involved in this pathway, we confirmed these results by further experimentation. SEW 2871, a selective agonist of S1PR1 (EC_50_ = 13 nM) failed to elicit an increase in IL-8 release above vehicle treated cells (Fig. 2 A). To ensure that CAY 10444 was active as a S1PR3 inhibitor at 10 µM, we cultured human airway smooth muscle cells on glass coverslips and measured their intracellular calcium responses to S1P (1 µM) stimulation. Pre-treating the cells with CAY 10444 abolished the S1P induced calcium responses in these cells, suggesting that the inhibitor was indeed active at these concentrations (Fig. 2 E). CAY 10444 treatment appeared to cause an increase in resting intracellular calcium levels in human airway smooth muscle cells, again indicating the biological effect of CAY 10444 at a concentration of 10 µM (Fig. 2 D).

### NF-κB Mediates S1P Induced IL-8 Release

**Figure 3:**
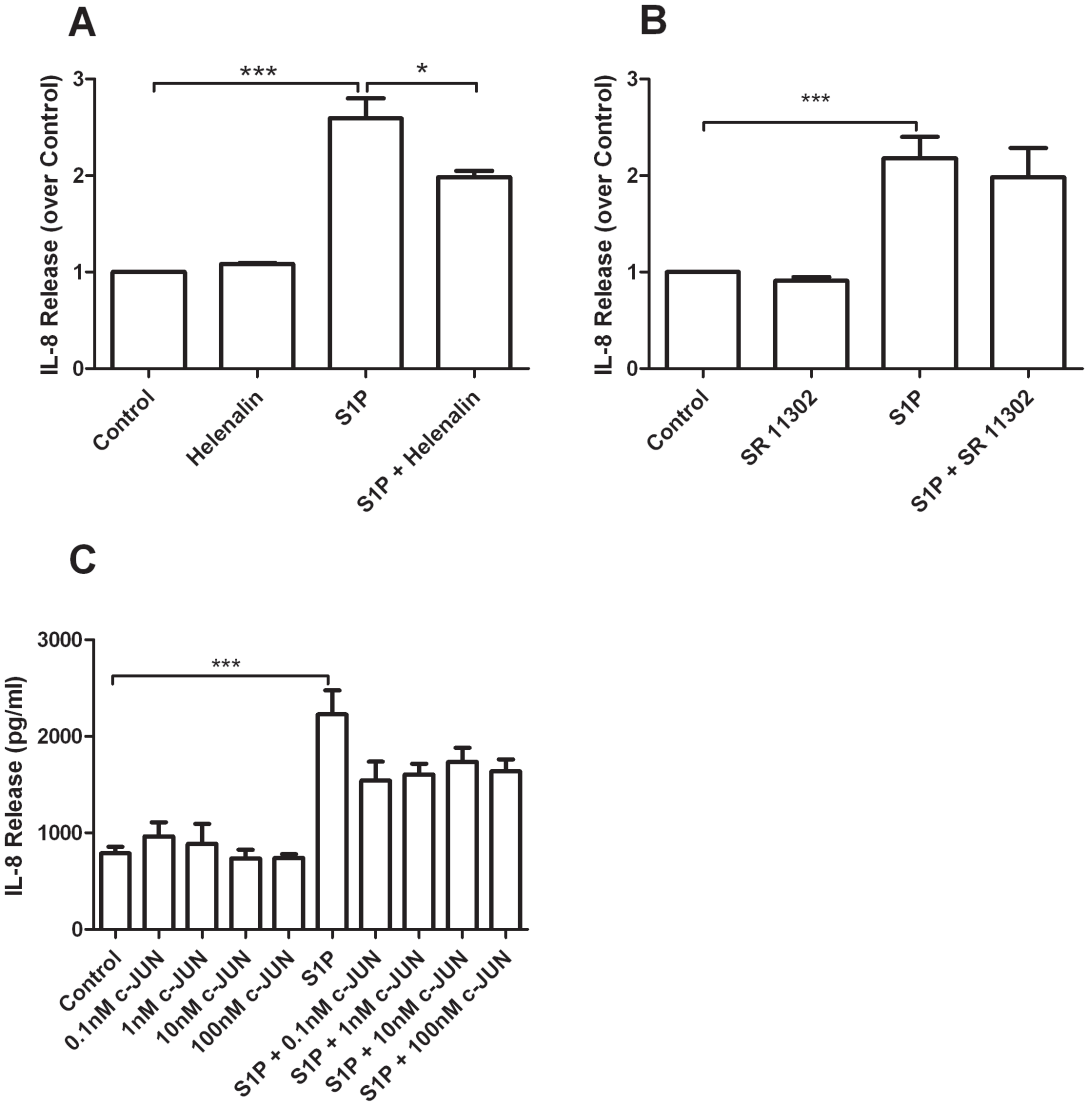
S1P induced IL-8 release is mediated by NF-κB in BEAS-2B cells. BEAS-2B cells were pretreated with (A) Helenalin (n = 3), (B) SR 11302 (n = 3), or c-JUN peptide (n = 3) inhibitors of NF-κB, AP-1 and AP-1 respectively, for 30 minutes and then stimulated with S1P for 4 hours. Culture supernatants were analyzed for IL-8 concentration by ELISA. Data are representative of means+SE. ANOVA with Tukey post hoc pairwise comparisons. *P<0.05, ***P<0.001.

IL-8 synthesis is driven primarily by the transcription factors NF-κB and AP-1. We wished to assess if pharmacological inhibition of these transcription factors affected IL-8 release. BEAS-2B cells were pre-treated for 30 minutes with Helenalin or SR11302, specific inhibitors of NF-κB [16] and AP-1 [17] respectively, prior to S1P stimulation for 4 hours. Pre-treatment with Helenalin but not with SR11302 inhibited IL-8 release (Fig. 3 A–B). To further confirm that AP-1 is not involved in S1P induced IL-8 release, we pre-treated cells with a cell permeant peptide fragment of the AP-1 monomer c-JUN. The peptide fragment contained amino acids 33–57 of the JNK binding domain and disrupts the interaction of JNK and c-JUN, preventing c-JUN phosphorylation. The peptide inhibitor did not significantly reduce S1P induced IL-8 release (Fig. 3 C). However there was a trend towards a decrease in IL-8 secretion, raising the possibility that AP-1 may play a minor role in S1P induced IL-8 release. We conclude from these experiments that NF-κB is the dominant transcription factor determining the magnitude of S1P induced IL-8 release.

### S1P Induced NF-κB Activity is Dependent on S1PR2

**Figure 4:**
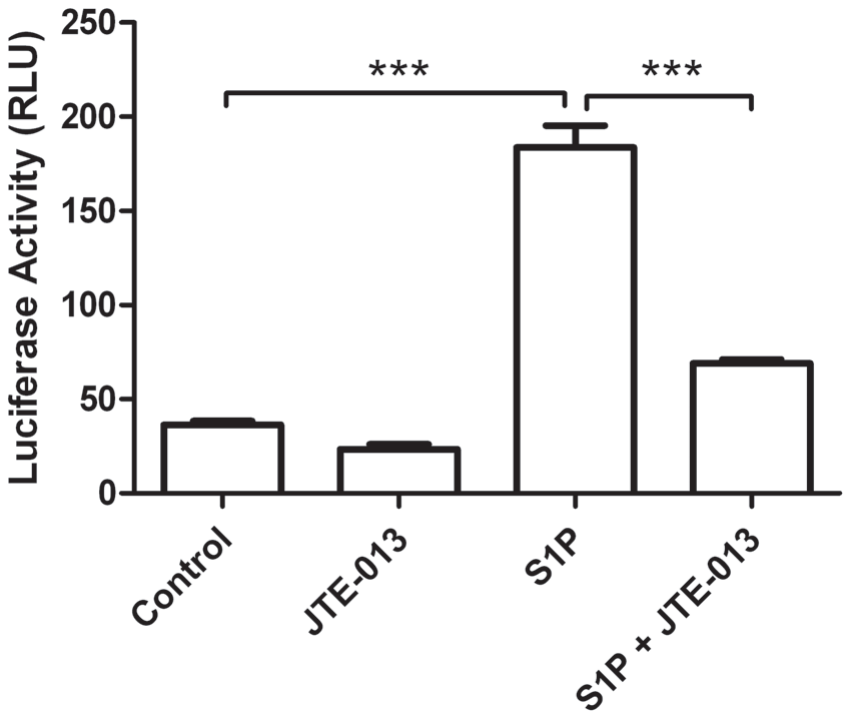
S1P induced Nf-κB activity is mediated by S1PR2 in BEAS-2B cells. Nf-κB luciferase reporter BEAS-2B cells were pretreated with S1PR2 inhibitor JTE 013 for 30 minutes before stimulation with S1P for 4 hours (n = 3). Cell lysates were analyzed for luciferase activity by Tecan iControl plate reader. Data are representative of means+SE. ANOVA with Tukey post hoc pairwise comparisons. ***P<0.001.

To further confirm that NF-κB drives S1P induced IL-8 release, we used a BEAS-2B NF-κB luciferase reporter cell line. Stimulation of the reporter cells with S1P for 4 hours induced a significant increase in luciferase activity (Fig. 4). Since S1P induced IL-8 release was dependent on S1PR2, we evaluated whether S1P induced NF-κB activation was also dependent on S1PR2. Pre-treatment of the BEAS-2B NF-κB luciferase reporter cells with JTE-013 significantly reduced the S1P induced luciferase activity (Fig. 4). These results indicate that S1P mediates its activation of NF-κB via S1PR2.

### S1P Induced IL-8 Release is not Dependent on the Epidermal Growth Factor Receptor

**Figure 5:**
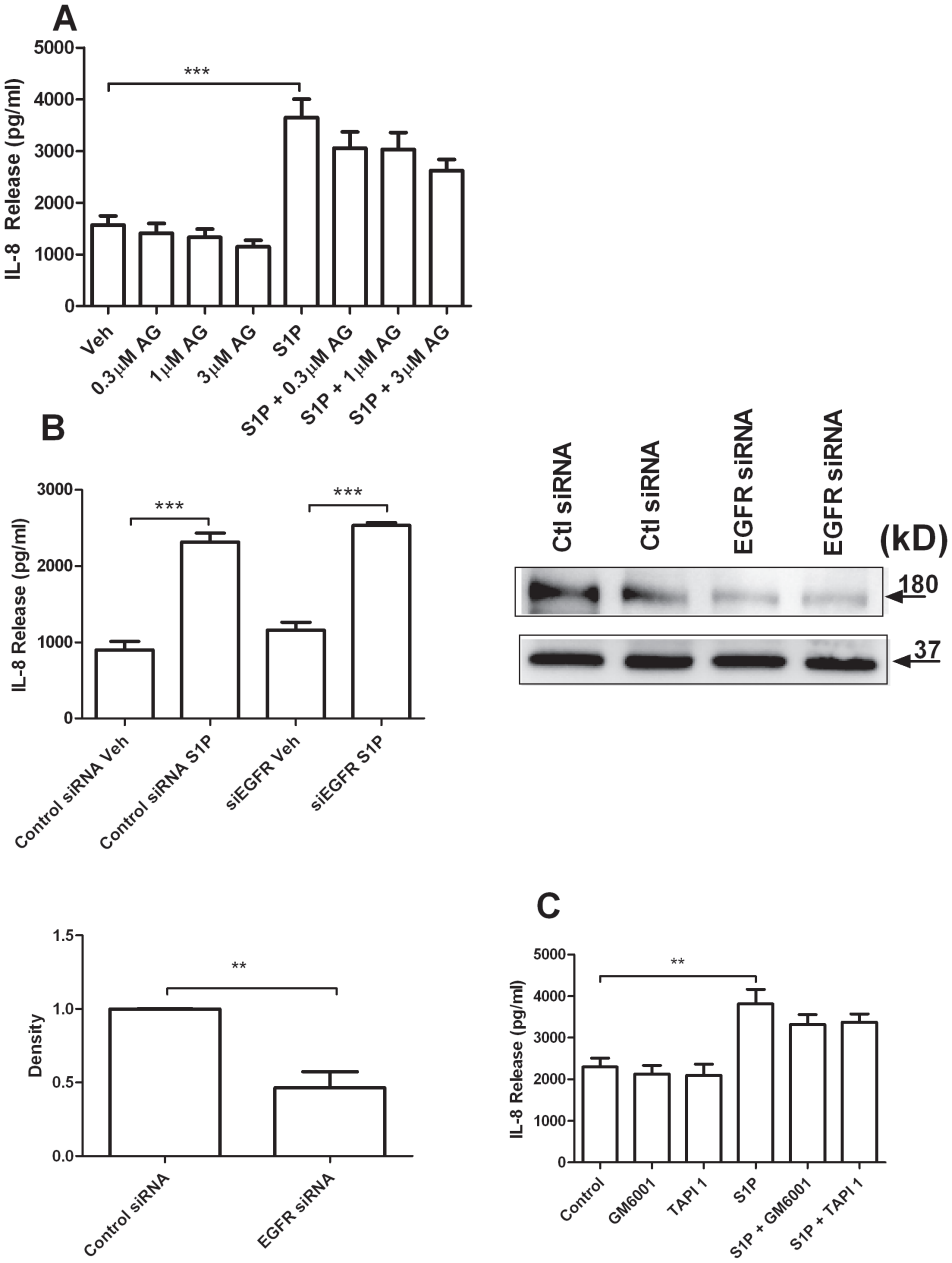
S1P induced IL-8 release is not dependent on the EGFR in BEAS-2B cells. (A) BEAS-2B cells were pretreated for 30 minutes with the specific EGFR inhibitor AG 1478 and then stimulated with S1P for 4 hours (n = 7). Culture medium was assessed for IL-8 concentration by ELISA. (B) BEAS-2B cells transfected with control or EGFR specific siRNA were stimulated with S1P for 4 hours (n = 3). Culture medium was assessed for concentrations of IL-8 by ELISA (left). Knock-down efficacy is shown by western blot for total EGFR (170 kDa) with GAPDH loading control (37 kDa) (right). Quantification of total EGFR bands is shown (n = 4) (bottom left). (C) BEAS-2B cells were pretreated for 30 minutes with MMP inhibitors GM6001 or TAPI-1 and then stimulated with S1P for 4 hours (n = 3). Culture medium was assessed for IL-8 concentration by ELISA. Data are representative of means+SE. ANOVA with Tukey post hoc pairwise comparisons. *P<0.05, **P<0.01, ***P<0.001.

Since activation of the EGFR can be upstream of IL-8 release [13] we wished to explore if EGFR transactivation is induced by S1P stimulation. Pretreatment with EGFR tyrosine kinase inhibitor AG1478 for 30 minutes prior to S1P stimulation failed to reduce S1P induced IL-8 release at reasonable concentrations for EGFR inhibition (Fig. 5 A). To confirm these results we used siRNA against the EGFR and knocked down 54% of the constitutively expressed protein (Fig. 5 B). Upon S1P stimulation, there was no difference in IL-8 release between siEGFR and scrambled siRNA groups (Fig. 5 B). We conclude that the transactivation of the EGFR is not involved in mediating S1P induced IL-8 secretion from airway epithelial cells. In support of this conclusion, we failed to measure an increase in phosphorylation of tyrosine-845 by western blot after stimulation with S1P (data not shown).

We sought further supportive evidence that EGFR transactivation does not occur in S1P induced IL-8 release exists. Matrix metalloproteinases (MMPs) often play a role in transactivating the EGFR by causing the release of pro-forms of EGFR ligands [13], [18], [19]. GM6001 and TAPI-1, inhibitors of MMPs did not inhibit S1P induced IL-8 release (Fig. 5 D) pointing towards an EGFR-independent mechanism.

### S1P Induced IL-8 Release is not Mediated by Reactive Oxygen Species

**Figure 6:**
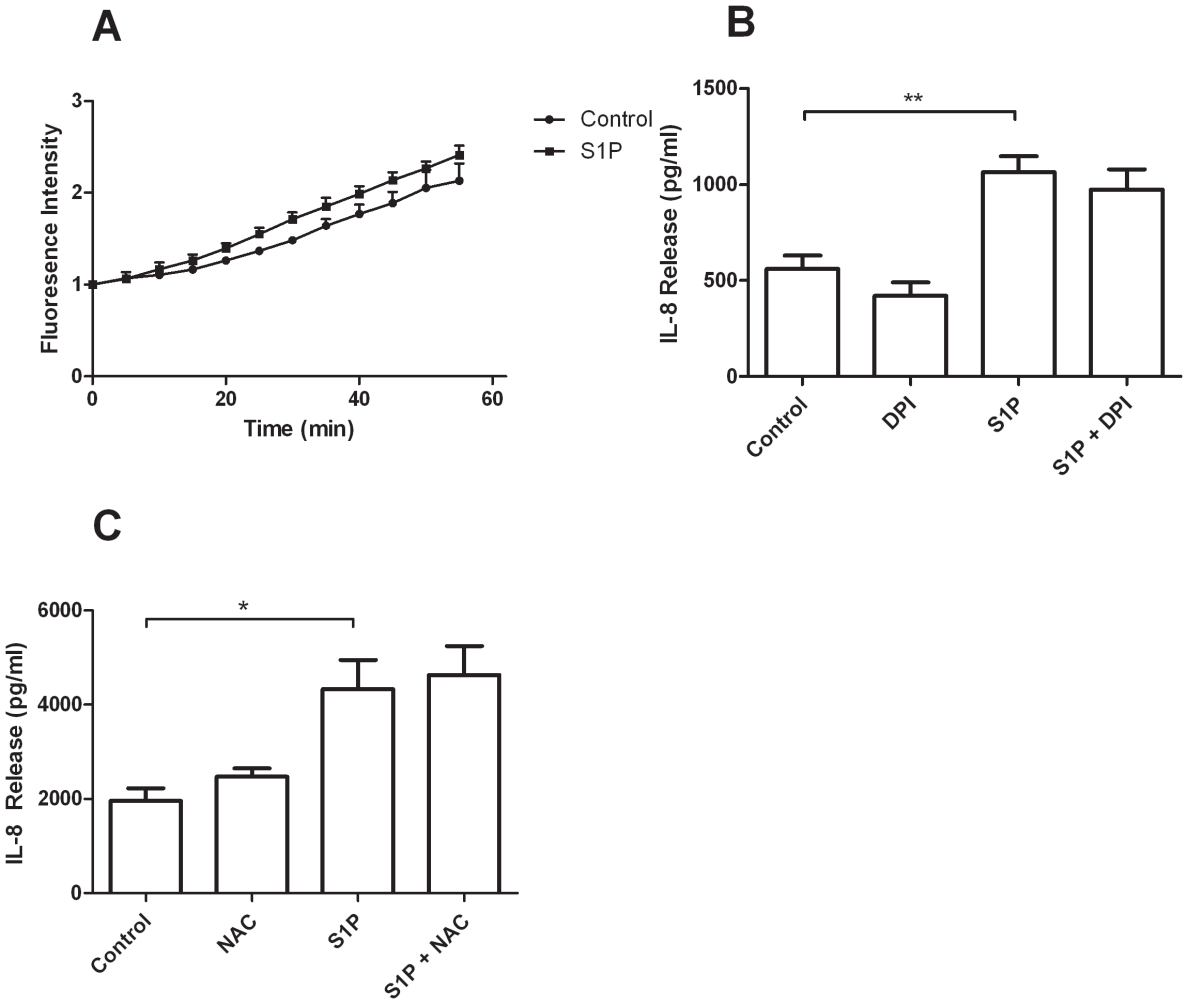
S1P induced IL-8 release is not dependent on the production of reactive oxygen species in BEAS-2B cells. (A) BEAS-2B cells were incubated for 30 minutes with 10 µM DCFH-DA. Free probe was washed with Hank’s buffer and baseline fluorescence was measured at 530 nm. Cells were then stimulated with S1P or vehicle and fluorescence intensity was measured every 5 minutes for one hour (n = 3). Analysis using repeated measures ANOVA revealed no difference between the two curves. (B) BEAS-2B cells were pretreated for 30 minutes with the general antioxidant N-acetyl cysteine (NAC) and then stimulated with S1P for 4 hours (n = 5). Culture medium was assessed for IL-8 concentration by ELISA. (C) BEAS-2B cells were pretreated for 30 minutes with the NADPH oxidase inhibitor DPI and then stimulated with S1P for 4 hours (n = 5). Culture medium was assessed for IL-8 concentration by ELISA. (n = 5). Data are representative of means+SE. ANOVA with Tukey post hoc pairwise comparisons *P<0.05, **P<0.01.

To determine if the generation of ROS mediates S1P induced IL-8 release, we measured the production of ROS in BEAS-2B cells using the fluorescent ROS probe DCFH. S1P did not induce significant increases in fluorescence (Fig. 6 A). To further confirm that S1P does not induce IL-8 release through the production of ROS, we incubated BEAS-2B cells with the ROS scavenger N-acetyl cysteine for 30 minutes and then stimulated with S1P for 4 hours. ELISA analysis for IL-8 revealed that pretreatment with the antioxidant NAC is unable to decrease S1P induced IL-8 release (Fig. 6 B). Pretreatment for 30 minutes with the general NADPH oxidase inhibitor DPI was also unable to inhibit S1P induced IL-8 release (Fig. 6 C). Taken together, these results indicate that S1P induced IL-8 release is not dependent on the generation of ROS.

## Discussion

The purpose of this study was to evaluate the potential role of various S1P receptors and the mechanisms of transduction of the pro-inflammatory response of the airway epithelium to S1P. S1P induced IL-8 secretion represents an important avenue for the recruitment of neutrophils to the airways, particularly in asthma. Inhibition of this phenomenon could lead to improvement of lung function in severe asthmatic patients, and improve their symptoms. We have identified S1PR2 as a receptor responsible for transducing the S1P signal from the cell exterior, causing IL-8 secretion from BEAS-2B cells.

S1P is an important lipid mediator that has been implicated in a number of biological processes. It has been shown to be present in increased concentrations in the airways of asthmatic subjects, where it may act on the lining epithelial cells. S1P has been shown previously to trigger IL-8 secretion by cultured epithelial cells [11], [12]. To confirm these previous findings, we stimulated BEAS-2B cells with various concentrations of S1P to construct a concentration-response relationship. Our results demonstrate that 1 µM S1P is sufficient to cause a significant increase in IL-8 secretion, consistent with previously published data [11].

A study by Milara et al. examined the effect of S1P on IL-8 secretion from another airway epithelial cell line, A549. Milara et al. demonstrated that S1P induced the release of IL-8 from A549 cells at later time points than we show here in BEAS-2B cells. Also, A549 cells secreted much more IL-8 at basal levels than did BEAS-2B cells in this study. The conditioned medium from A549 cells recruited neutrophils in a Boyden chamber assay. [20] Milara et al. also implicated the crucial role of phospholipase D signaling in S1P induced IL-8 release in A549 cells, which has been described in BEAS2-B cells by Wang et al. [12].

Following identification of S1PR2 and NF-κB as important mediators of S1P induced IL-8 release, the question of how this receptor signals to the transcription factor arises. Others have described the effect of S1PR2 blockade on various physiological processes; for example antagonizing S1PR2 in human bronchial epithelial cells inhibits the extrusion of apoptotic cells [21]. S1P receptor G protein coupling is complex, with S1PR2 coupling to a variety of Gα subunits including Gα_s_, Gα_i_, Gα_12/13_, Gα_q_ and G_0_ [22]. Increased cell survival mediated by NF-κB stimulation by S1P in HeLa cells is transduced through S1PR2 coupling with Gα_i_ [23]. Furthermore, evidence exists that S1P induced IL-8 secretion is Gα_i_ and Rho dependent which in turn drives phospholipase D activation [11]. Lipopolysaccharide (LPS), a ligand for toll-like receptor 4 (TLR4), stimulates RhoA, which then activates NF-κB to release IL-8 in cervical stromal cells [24]. In the endothelium, activation of S1PR2 leads to activation of the small GTPase Rho [25]. Recently it was shown that LPS and tumor necrosis factor-alpha (TNF-α)-induced endothelial inflammation is mediated by S1PR2, coupling to the transcription factor NF-κB. [26] This literature supports the model that S1PR2 activation can drive NF-κB to up-regulate IL-8 synthesis and secretion in airway epithelial cells.

S1P administration in vivo to mice has been shown to increase airway reactivity to methacholine challenge, increase airway eosinophil recruitment, and increase interleukin (IL)-4, IL-13 and IL-17 in the BAL. In this study, mice were treated with S1P subcutaneously before analysis of airway function. [27] This study did not examine the production of chemokines associated with airway neutrophil recruitment, but adds strength to the rationale for studying this molecule in the context of lung disease by demonstrating that S1P itself can induce airway hyperesponsiveness. Inhibition of mouse lung S1PR2 with JTE-013 inhibited S1P induced pulmonary vasoconstriction, another S1PR2/Rho kinase dependent phenomenon [28].

S1P can induce bronchial smooth muscle contraction which has been shown to be dependent on S1PR2 and rho kinase [29]. Rho kinase has also been implicated in airway smooth muscle contraction from ovalbumin challenged mice, linking rho kinase activity to an allergic model of asthma [30]. This literature provides evidence that S1PR2/Rho kinase signaling could drive asthma pathogenesis not only by augmenting neutrophilic inflammation, but also by increasing airway smooth muscle contractility. Inhibition of S1PR2 in the lung of severe asthmatics could therefore cause relaxation of the bronchial smooth muscle and resolve inflammation strengthening the rationale for the use of JTE-013 as a pharmacological tool for the treatment of non-eosinophilic asthma.

Oxidative stress is an important mediator of other GPCR pathways [31], [32] and is involved in asthma pathogenesis [33], [34]. The NADPH oxidase enzyme is a large producer of oxidative stress in airway epithelial cells. A functional NADPH oxidase has been demonstrated to be essential in NF-κB activation in *Pseudomonas aeruginosa* infected mouse phagocytic leukocytes [35]. It has been previously shown that oxidative stress from NADPH oxidase activation can transactivate the EGFR [14]. ROS generation within the cell is able to inactivate protein tyrosine phosphatases by oxidation of cysteine residues, shifting the EGFR to a more activated state [36]. Specific inhibition of the NADPH oxidase, or use of a general anti-oxidant failed to inhibit S1P induced IL-8 secretion, nor did we measure any significant increase in cellular oxidative stress after S1P administration. In contrast hematopoietic progenitor cells egress the bone marrow under the influence of S1P in a ROS dependent manner via signaling through S1PR1 [37]. Similarly cardiac fibrosis is mediated by S1PR3 and oxidative stress in sphingosine kinase 1 transgenic mice [38]. Since the signal for IL-8 release in our experiments was S1PR2-dependent, it suggests that the role of ROS in S1P receptor signaling may be receptor specific.

We have tested and rejected the hypothesis that EGFR transactivation mediates S1P induced IL-8 release in BEAS-B cells. Pharmacological inhibition of the EGFR with the tyrosine kinase inhibitor AG 1478 failed to inhibit S1P induced IL-8 secretion at concentrations appropriate for selective EGFR inhibition. The IC_50_ of AG1478 for inhibition of the EGFR is 3 nM [39] Knockdown of the EGFR using small interfering RNA failed inhibit IL-8 release despite a 54% reduction in EGFR protein. Other studies have noted that AG 1478 may have non-specific effects [40], [41]. We also failed to detect any increases in phosphorylation of tyrosine 845 of the EGFR after stimulation with S1P, a further indication that transactivation of the EGFR by S1P does not occur. This demonstrates that there is not a common mechanism of IL-8 secretion induced by either S1P or LTD_4_ in BEAS-2B cells.

This study has shown that, *in vitro*, pharmacological inhibition of S1PR2 decreases S1P induced IL-8 release from BEAS-2B cells, and that S1PR2 is upstream of NF-κB in this phenomenon. Future translational work *in vivo* using S1PR2 antagonists in animal models of asthma will be of great interest.
